# Supplementary material for: Creating a bacterium that forms eukaryotic nucleosome core particles
Source: Nat Commun. 2024 Sep 27;15:8283. doi: 10.1038/s41467-024-52484-2 (PMC11436726; doi:10.1038/s41467-024-52484-2)
Supplement: Supplementary file 1 — Supplementary Information [file 41467_2024_52484_MOESM1_ESM.doc]

**Supplementary Information for**

Creating a bacterium that forms eukaryotic nucleosome core particles

Xinyun Jing1,8, Niubing Zhang1,2,8, Xiaojuan Zhou1,3,8, Ping Chen1,4,8, Jie Gong1,3,8, Kaixiang Zhang1,2,3, Xueting Wu1, Wenjuan Cai5, Bang-Ce Ye2, Pei Hao3,4, Guo-ping Zhao1,6,7, Sheng Yang1,3, Xuan Li1,3,*

1Key Laboratory of Synthetic Biology, Key Laboratory of Plant Design, CAS Center for Excellence in Molecular Plant Sciences, Chinese Academy of Sciences, Shanghai 200032, China

2State Key Laboratory of Bioreactor Engineering, East China University of Science and Technology, Shanghai 200237, China

3University of Chinese Academy of Sciences, Beijing 100039, China

4Key Laboratory of Molecular Virology and Immunology, Shanghai Institute of Immunity and Infection, Chinese Academy of Sciences, Shanghai 200031, China

5Core Facility Center, CAS Center for Excellence in Molecular Plant Sciences, Chinese Academy of Sciences, Shanghai 200032, China

6CAS Key Laboratory of Quantitative Engineering Biology, Shenzhen Institute of Synthetic Biology, Shenzhen Institutes of Advanced Technology, Chinese Academy of Sciences, Shenzhen 518055, China

7Key Laboratory of Systems Health Science of Zhejiang Province, School of Life Science, Hangzhou Institute for Advanced Study, University of Chinese Academy of Sciences, Hangzhou, China.

8These authors contributed equally

* Corresponding author

Email: [lixuan@sippe.ac.cn](mailto:lixuan@sippe.ac.cn) (Xuan Li)

**This file contains Supplementary Figures 1 to 5.­­**


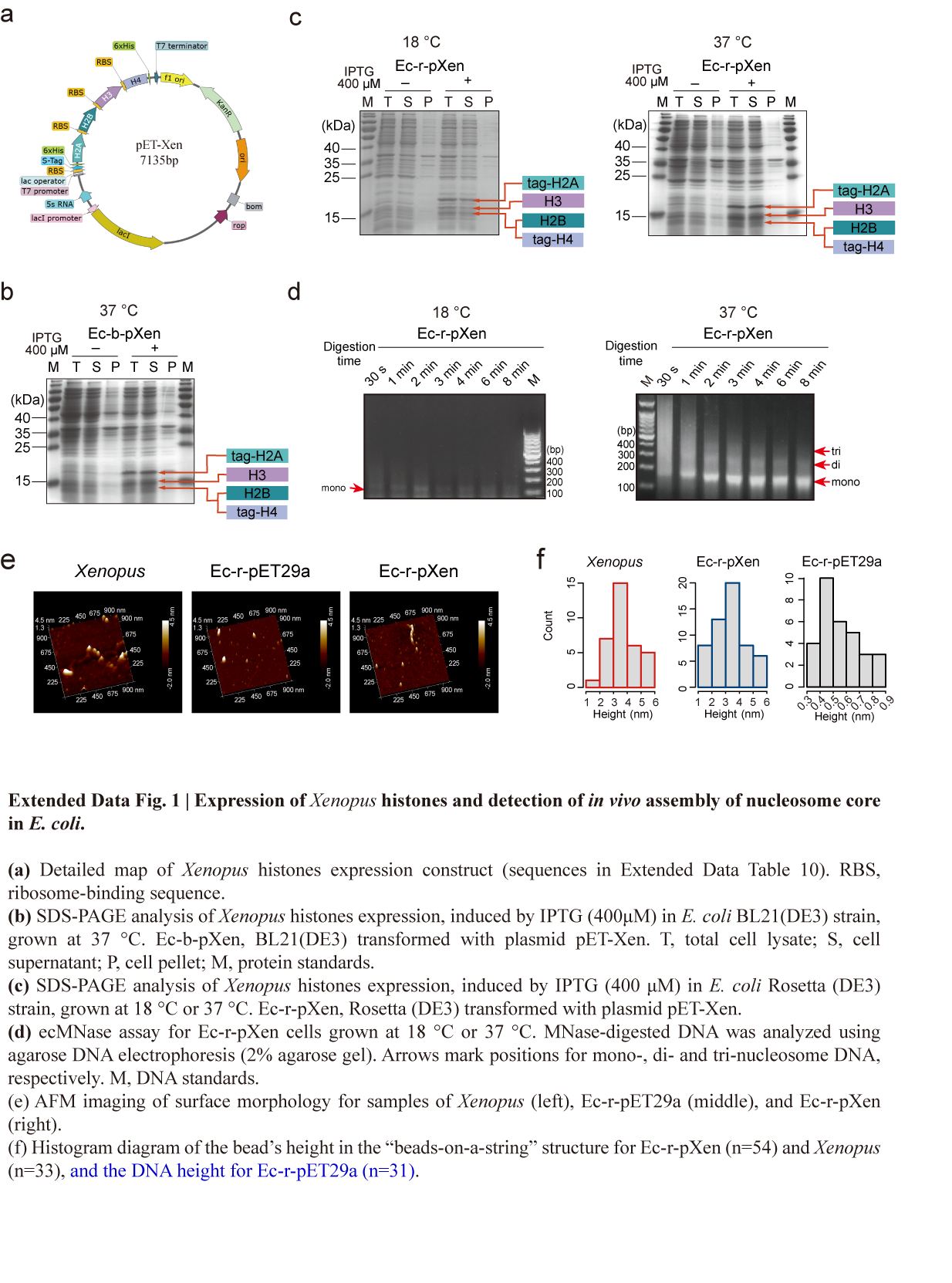
­**Supplementary Figure 1 | Expression of *Xenopus* histones and detection of *in vivo* assembly of nucleosome core in *E. coli*. a,** Detailed map of *Xenopus* histones expression construct (sequences in Supplementary Data 8). RBS, ribosome-binding sequence. **b,** SDS-PAGE analysis of *Xenopus* histones expression, induced by IPTG (400μM) in *E. coli* BL21(DE3) strain, grown at 37 °C. Ec-b-pXen, BL21(DE3) transformed with plasmid pET-Xen. T, total cell lysate; S, cell supernatant; P, cell pellet; M, protein standards. The figure is representative of three independent experiments. **c,** SDS-PAGE analysis of *Xenopus* histones expression, induced by IPTG (400 μM) in *E. coli* Rosetta(DE3) strain, grown at 18 °C or 37 °C. Ec-r-pXen, Rosetta (DE3) transformed with plasmid pET-Xen. The figure is representative of three independent experiments. **d,** ecMNase assay for Ec-r-pXen cells grown at 18 °C or 37 °C. MNase-digested DNA was analyzed using agarose DNA electrophoresis (2% agarose gel). Arrows mark positions for mono-, di- and tri-nucleosome DNA, respectively. M, DNA standards. The complete gel pieces are provided in Supplementary Fig. 5. The figure is representative of three independent experiments. **e,** AFM imaging of surface morphology for samples of *Xenopus* (left), Ec-r-pET29a (middle), and Ec-r-pXen (right). **f,** Histogram diagram of the bead’s height in the “beads-on-a-string” structure for Ec-r-pXen (n=54), and *Xenopus* (n=33), and the DNA height for Ec-r-pET29a (n=31). Source data are provided as a Source Data file.

**
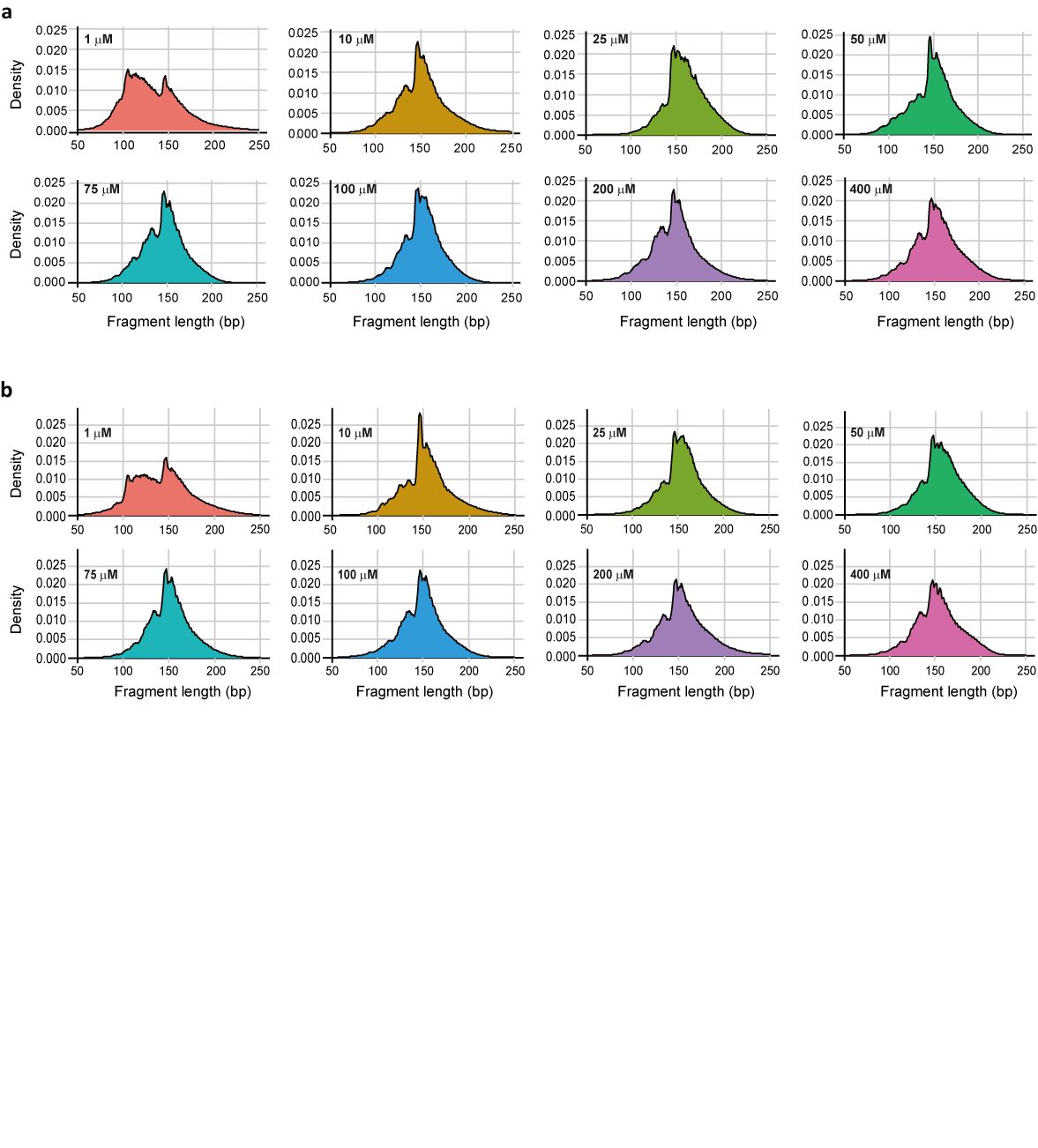
**

**Supplementary Figure 2 | Length distribution of ecMNase-seq reads from Ec-r-pXen cells grown at 37** °C **and treated with IPTG at different concentrations. a** and **b** represent datasets of two independent experiments. The ecMNase-seq reads contain two populations separated by a valley at ~140 bp. The larger-size population had a major peak at 146-147 bp. The sub-nucleosomal size populations sometimes display minor bumps at 105-106, 115-116, 125-126, or 135-136 bp, representing the products from internal digestion of mono-nucleosome DNA. Source data are provided as a Source Data file.


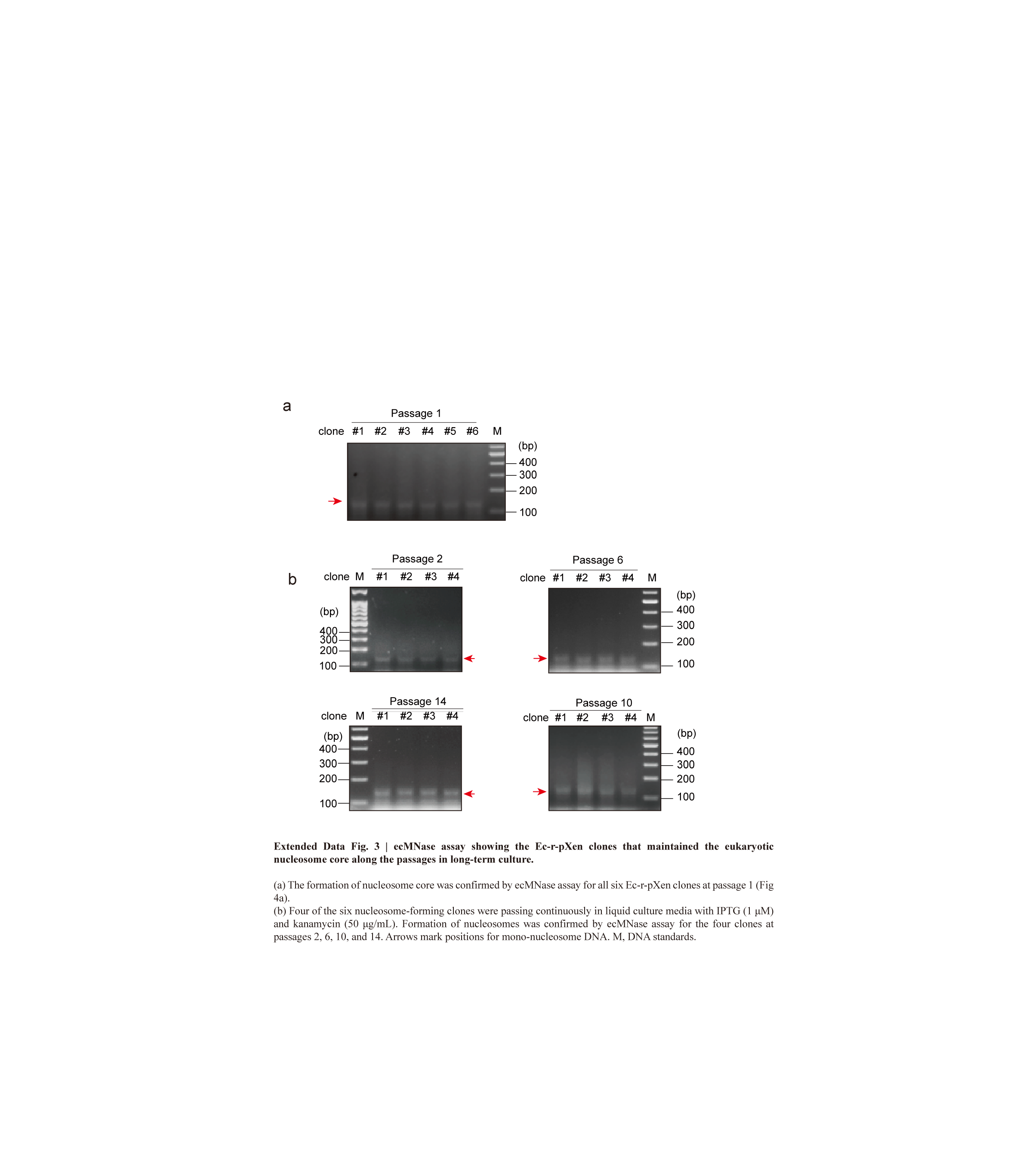


**Supplementary Figure 3 | ecMNase assay showing the Ec-r-pXen clones that maintained the eukaryotic nucleosome core along the passages in long-term culture. a,** The formation of nucleosome core was confirmed by ecMNase assay for all six Ec-r-pXen clones at passage 1 (Fig. 4a). **b,** Four of the six nucleosome-forming clones were passing continuously in liquid culture media with IPTG (1 μM) and kanamycin (50 μg/mL). Formation of nucleosomes was confirmed by ecMNase assay for the four clones at passages 2, 6, 10, and 14. Arrows mark positions for mono-nucleosome DNA. M, DNA standards. The complete gel pieces were provided in Supplementary Fig. 5. Source data are provided as a Source Data file.


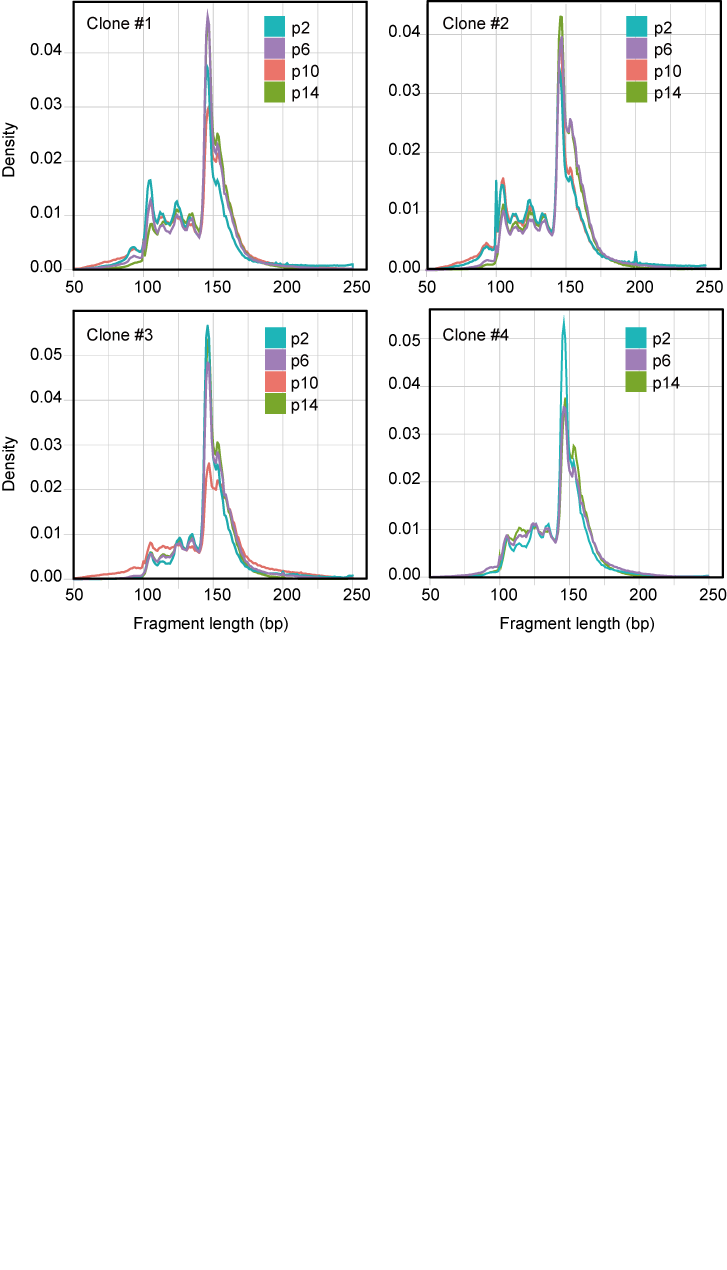


**Supplementary Figure 4 | Length distribution of ecMNase-seq reads from four Ec-r-pXen clones along the passages in long-term culture.** For each of the nucleosome-forming clones, their ecMNase-seq data displayed a consistent profile along passages, indicating the stabilized formation of eukaryotic nucleosomes in *E. coli* in the long-term growth experiment. The minor peaks at 105-106, 115-116, 125-126, and 135-136 bp were more apparent in comparison to those of 1 μM-IPTG induction experiments. P2, P6, P10 and P14 represent passage #2, #6, #10 and #14, respectively. Source data are provided as a Source Data file.

a


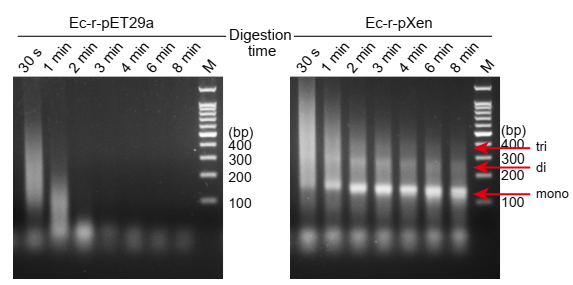


b


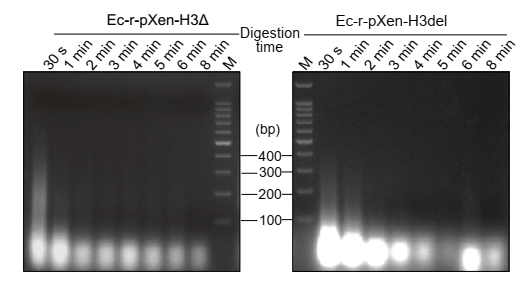


c


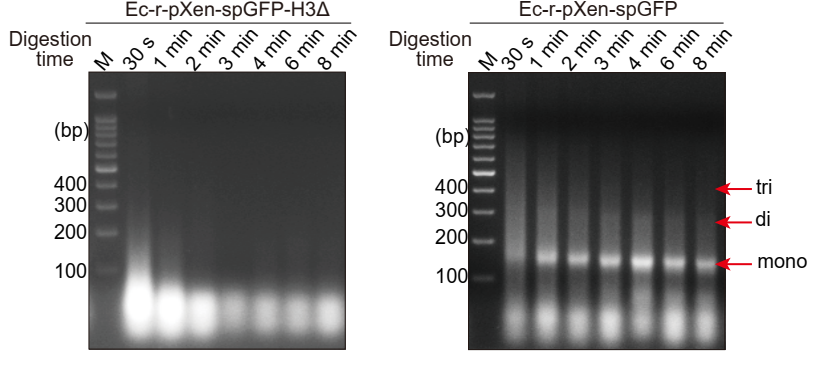
­­

d


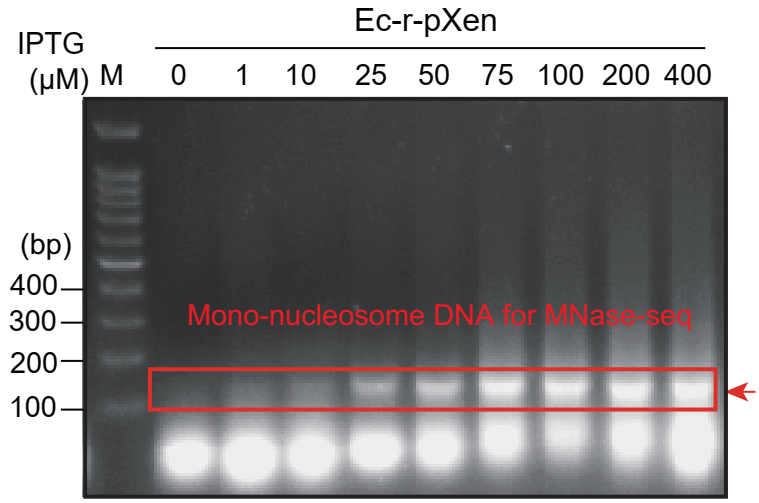


e


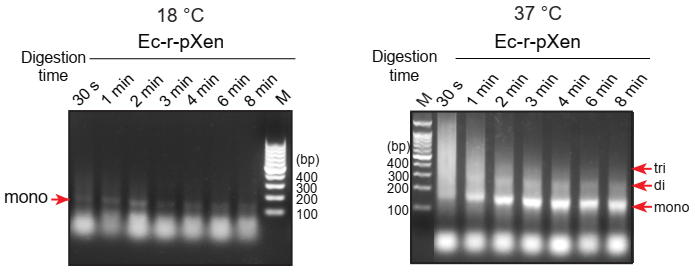


f


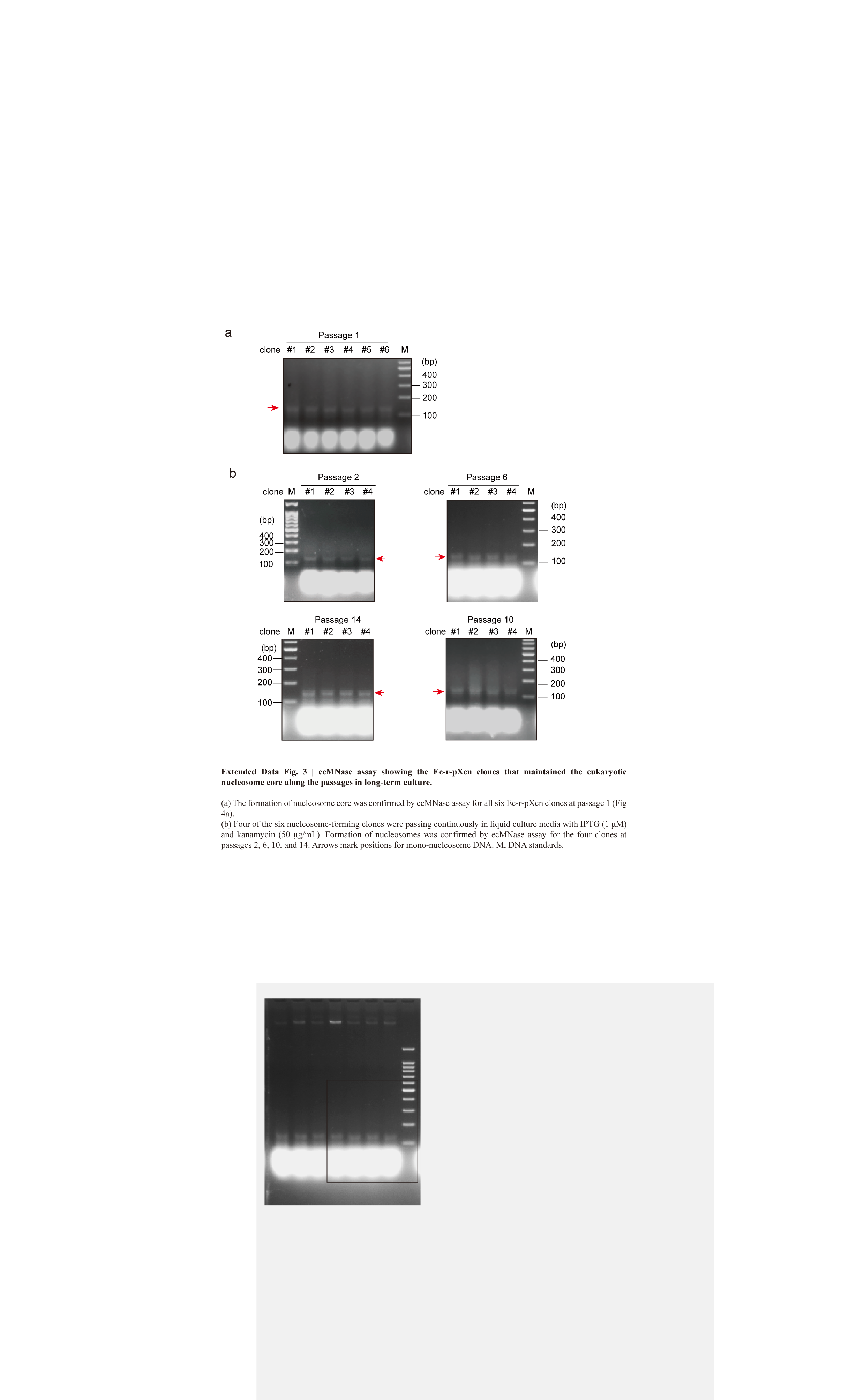


g

­
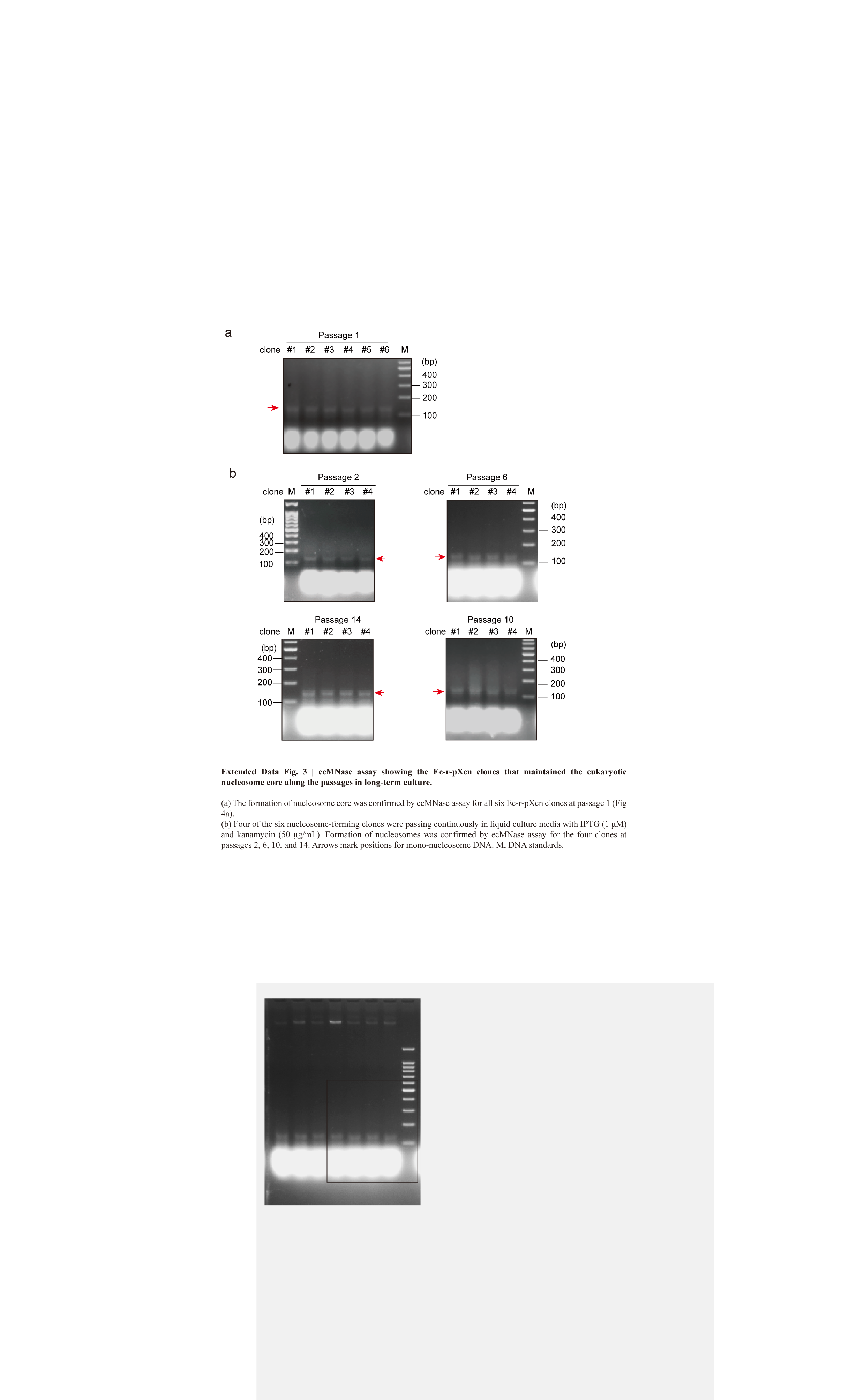


**Supplementary Figure 5 | The complete gel pieces related to Fig. 1d, Fig. 1h, Fig. 2c, Fig. 3c, Supplementary Fig. 1d, Supplementary Fig. 3a, and Supplementary Fig. 3b.**

**a,** The complete gel piece related to Fig. 1d. **b,** The complete gel piece related to Fig. 1h. **c**, The complete gel piece related to Fig. 2c. **d**, The complete gel piece related to Fig. 3c. **e**, The complete gel piece related to Supplementary Fig. 1d. **f**, The complete gel piece related to Supplementary Fig. 3a. **g**, The complete gel piece related to Supplementary. 3b. Source data are provided as a Source Data file.
