# Supplementary material for: Creating a bacterium that forms eukaryotic nucleosome core particles
Source: Nat Commun. 2024 Sep 27;15:8283. doi: 10.1038/s41467-024-52484-2 (PMC11436726; doi:10.1038/s41467-024-52484-2)
Supplement: Supplementary file 3 — Description of Additional Supplementary Files [file 41467_2024_52484_MOESM3_ESM.pdf]

Supplementary data 1: Number of nucleosome peaks and sequence quantities used for peak calling in the IPTG-titration experiments.

Supplementary Data 2: Transcriptional level across four different clones at varying passages.

Supplementary Data 3: Number of nucleosome peaks called for different passages during long-term growth.

Supplementary Data 4: Differentially expressed genes (DEGs) in the nucleosome-forming strain (Ec-r-pXen) compared to the control strain (Ec-r-pET29a).

Supplementary Data 5: SNPs from clone #1, 2, 3, and 4 throughout the passages 2, 6, 10, and 14 (labled as p2, p6, p10, and p14), identified by whole genome-seq (WGS) with allele frequency (AF) >= 10% and not present in the control strain (Ec-r-pET29a).

Supplementary Data 6: Proteins identified in strain Ec-r-pXen treated with 1 μM IPTG using liquid chromatography-tandem mass spectrometry coupled with multiple reactions monitoring (LC-MRM-MS).

Supplementary Data 7: Proteins identified in strain Ec-r-pET29a treated with 1 μM IPTG using liquid chromatography-tandem mass spectrometry coupled with multiple reactions monitoring (LC-MRM-MS).

Supplementary Data 8: Gene sequences of RBS-H2A-RBS-H2B-RBS-H3-RBS-H4 in the context of pET-Xen plasmid, under the promoter of T7 and T7 terminator. Nucleotides for RBS, pink letters; nucleotides for S-tag, underlined blue letters; nucleotides for His6-tag, wave-underlined letters; nucleotides for H2A, blue letters; nucleotides for H2B, red letters; nucleotides for H3, brown letters; nucleotides for H4, purple letters.

Supplementary Data 9: A list of primers.

Supplementary Data 10: Gene sequences of RBS-(GFP1-9-H2A)-RBS-(H2B-GFP10)-RBS-H3-RBS-(GFP11-14-H4) in the context of pET-Xen-spGFP plasmid, under the promoter of T7 and T7 terminator. Nucleotides for RBS, pink letters; nucleotides for H2A, blue letters; nucleotides for H2B, red letters; nucleotides for H3, brown letters; nucleotides for H4, purple letters, nucleotides for sfGFP, green letters; linker, grey letters.

Supplementary Data 11: Gene sequences of RBS-(GFP1-9-H2A)-RBS-(H2B-GFP10)-RBS-H3Δ(101-132)-RBS-(GFP11-14-H4) in the context of pET-Xen-spGFP-H3Δ plasmid, under the promoter of T7 and T7

terminator. Nucleotides for RBS, pink letters; nucleotides for H2A, blue letters; nucleotides for H2B, red letters; nucleotides for H3, green letters; nucleotides for H4, purple letters, nucleotides for sfGFP, green letters; linker, grey letters.

Supplementary Data 12: ecMNase-seq data files for IPTG-titration and long-term culture experiments. They have been deposited to NCBI SRA.

Supplementary Data 13: mRNA-seq data files for long-term growth experiments. They have been deposited to NCBI SRA.

Supplementary Data 14: Whole genome-seq data files for long-term growth experiments. They have been deposited to NCBI SRA.

Supplementary Data 15: Duration of culture time before the photos were taken for different conditions in Fig. 6
